# Discovery of Dihydrophaseic Acid Glucosides from the Florets of *Carthamus tinctorius*

**DOI:** 10.3390/plants9070858

**Published:** 2020-07-07

**Authors:** Su Cheol Baek, Bum Soo Lee, Sang Ah Yi, Jae Sik Yu, Jaecheol Lee, Yoon-Joo Ko, Changhyun Pang, Ki Hyun Kim

**Affiliations:** 1 School of Pharmacy, Sungkyunkwan University, Suwon 16419, Korea; schii513@daum.net (S.C.B.); kosboybs@naver.com (B.S.L.); angelna1023@hanmail.net (S.A.Y.); jsyu@bu.edu (J.S.Y.); jaecheol@skku.edu (J.L.); 2 Laboratory of Nuclear Magnetic Resonance, National Center for Inter-University Research Facilities (NCIRF), Seoul National University, Gwanak-gu, Seoul 08826, Korea; yjko@snu.ac.kr; 3 School of Chemical Engineering, Sungkyunkwan University, Suwon 16419, Korea

**Keywords:** *Carthamus tinctorius*, dihydrophaseic acid, electronic circular dichroism calculation, adipogenesis

## Abstract

*Carthamus tinctorius* L. (Compositae; safflower or Hong Hua) has been used in Korean traditional medicine for maintaining the homeostasis of body circulation. Phytochemical investigation was performed on the florets of *C. tinctorius* by liquid chromatography–mass spectrometry (LC/MS), which afforded two dihydrophaseic acid glucosides (**1** and **2**). Isolated compounds were structurally confirmed using a combination of spectroscopic methods including 1D and 2D nuclear magnetic resonance and high-resolution electrospray ionization mass spectroscopy. Their absolute configurations were established by quantum chemical electronic circular dichroism calculations and enzymatic hydrolysis. The anti-adipogenesis activity of the isolated compounds was evaluated using 3T3-L1 preadipocytes. Treatment with the dihydrophaseic acid glucoside (**1**) during adipocyte differentiation prevented the accumulation of lipid droplets and reduced the expression of adipogenic genes, *Fabp4* and *Adipsin*. However, compound **2** did not affect adipogenesis. Our study yielded a dihydrophaseic acid glucoside derived from *C. tinctorius,* which has potential advantages for treating obesity.

## 1. Introduction

Safflower, *Carthamus tinctorius* L., commonly known as Honghua, is a highly branched and thistle-like annual plant that is dispersed over regions of Korea, Japan, China, and Russia. This plant has been used in Chinese traditional medicine for various purposes, such as promoting blood circulation and menstruation, and alleviating pain [1]. *C. tinctorius* is also used as a nutraceutical in Korea owing to its effect in maintaining the homeostasis of body circulation [2]. In practice, the pistil of *C. tinctorius*, well-known as saffron, is widely used as a spice and an edible dying agent in various European dishes, including paella, risotto, and pasta. In fact, saffron has bFeen the world’s most expensive spice by weight for a long time, and its historical use has been proven to be used in the seventh century BC in an Assyrian botanical treatise [3]. Carthamin, one of the main components of *C. tinctorius*, has been used as a yellow, orange, and pink dying agent for clothes and fabrics. In earlier studies, phytochemical investigation of *C. tinctorius* revealed the presence of major compounds, such as quinochalcones—including carthamin, safflower yellow, and safflomin—flavonoids, alkaloids, and polyacetylenes [4]; moreover, some studies show that the polyacetylenes from *C. tinctorius* inhibit NO release, thus showing its potential use as a medicine for inflammatory diseases [5]. 

Plant hormones are natural products or organic substances with low-molecular-weight that can regulate essentially many physiological and developmental processes at micromolar (or even lower) concentrations during the life cycle of a plant [6]. Structurally diverse plant hormones have been reported; these include auxins, cytokinins, abscisic acids, gibberellins, ethylene, polyamines, jasmonates, salicylic acids, brassinosteroids, and dihydrophaseic acids [6]. Among these plant hormones, dihydrophaseic acids are apocarotenoid sesquiterpenoids, which are phaseic acids in which the keto group has been reduced to the corresponding alcohol, resulting in two hydroxy groups being positioned at the opposite sides of the six-membered ring. Previously, a dihydrophaseic acid has been reported from the flowers of *C. tinctorius*, and was identified as 4′-*O*-dihydrophaseic acid-*β*-D-glucopyranoside methyl ester [7]. Previous studies have shown that various plant hormones from biosynthetic pathways have been demonstrated to exhibit biological activities, including antimycobacterial [8], cytotoxic [9], and phytotoxic [10] effects.

**Figure 1:**
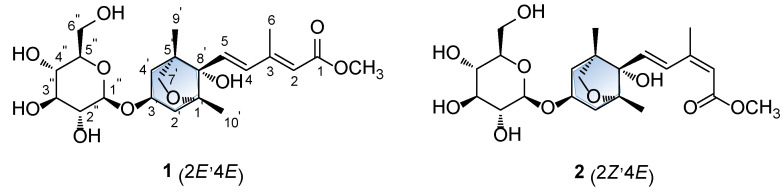
Structures of compound **1** and its geometric isomer **2**.

As a part of an ongoing effort to discover natural products that are structurally unique and/or biologically significant compounds from natural sources [11,12,13,14,15], phytochemical investigations on the methanol (MeOH) extract of the florets of *C. tinctorius* was undertaken. Chemical isolation and purification using liquid chromatography/mass spectroscopy (LC/MS) resulted in identification of two dihydrophaseic acid glucosides (**1** and **2**) (Figure 1). Their structures were determined by a combination of 1D and 2D nuclear magnetic resonance (NMR) spectroscopy and high-resolution (HR)-electrospray ionization-mass spectroscopy (ESI-MS) data. Furthermore, the absolute configurations of **1** and **2** were assigned by quantum chemical electronic circular dichroism (ECD) calculations and enzymatic hydrolysis. In addition, we evaluated the inhibitory effects of the isolates on the differentiation of 3T3-L1 adipocytes. In the present study, isolation and structural identification of **1** and **2** is demonstrated, as well as the significant inhibitory effects of compound **1** on lipid accumulation and adipogenic gene expression.

## 2. Results and Discussion

### 2.1. Isolation of Compounds

The dried florets of *C. tinctorius* were extracted using 80% aqueous MeOH under reflux to obtain the crude methanol extract, which was then subjected to solvent-partitioning with four solvents (hexane, dichloromethane, ethyl acetate, and *n*-butanol). Of the four solvent soluble fractions, EtOAc-soluble fraction was established with various column chromatography and high-performance liquid chromatography (HPLC) monitored with LC/MS analysis combined with our in-house UV library. The LC/MS-guided phytochemical analysis resulted in the isolation of two dihydrophaseic acid glucosides (**1** and **2)** (Figure 1).

### 2.2. Elucidation of Compound Structures

**Figure 2:**
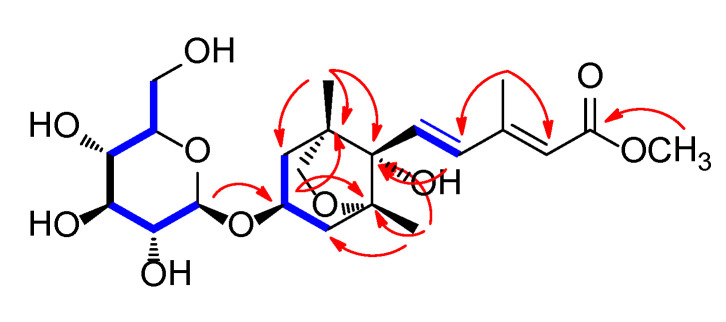
^1^H-^1^H COSY (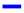) and key heteronuclear multiple bond correlation (HMBC) (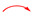) correlations for compound **1**.

Compound **1** was obtained as a white amorphous powder, and the molecular formula of **1** was established as C_22_H_34_O_10_ from the molecular ion peak [M + H]^+^ at *m/z* 459.2220 (calculated for C_22_H_35_O_10_ 459.2230) in positive-ion HR-ESIMS (Figure S1). The ^1^H (Figure S2) and ^13^C NMR data of **1** are presented in Table 1, and the ^13^C NMR signals were determined based on heteronuclear multiple bond correlation (HMBC) and heteronuclear single quantum coherence (HSQC). Interpretation of the ^1^H and ^13^C NMR data of **1** showed close similarities with those of (2Z,4*E*)-dihydrophaseic acid methyl ester-3-*O*-*β*-D-glucopyranoside (**2**), which was isolated in this study, and identified by comparing their NMR assignment with reported values [16,17] as well as by LC/MS analysis. However, differences in resonance were observed for the olefinic signals of H-4 (δ_H_ 6.66/δ_C_ 137.3 in **1**; δ_H_ 8.01/δ_C_ 131.5 in **2**) and H-6 (δ_H_ 2.34/δ_C_ 13.9 in **1**; δ_H_ 2.09/δ_C_ 20.8 in **2**) in the 3-methyl-penta-2,4-dienoic acid methyl ester group [7], which suggested that compound **1** may be a geometric isomer of **2**. Accordingly, the NMR data (Table 1) of compounds **1** and **2** were compared to verify their stereochemical difference. First, the complete planar structure of **1** was confirmed by 2D NMR [^1^H-^1^H COSY (Figure S3), HSQC (Figure S4), and HMBC (Figure S5)]. The connectivity of the bicycle moiety with the 3-methyl-penta-2,4-dienoic acid methyl ester was established on the HMBC cross-peaks from H-4 to C-8’ (Figure 2). The HMBC correlation of the anomeric proton at δ_H_ 4.37 (H-1’’) and C-3’ suggested evidence for the presence of a glucose group at C-3 (Figure 2). The complete gross structure of **1** was further elucidated based on the additional correlations in the ^1^H-^1^H COSY and HMBC spectra (Figure 2). Thus, compound **1** was determined to share the same planar structure with **2**. 

**Figure 3:**
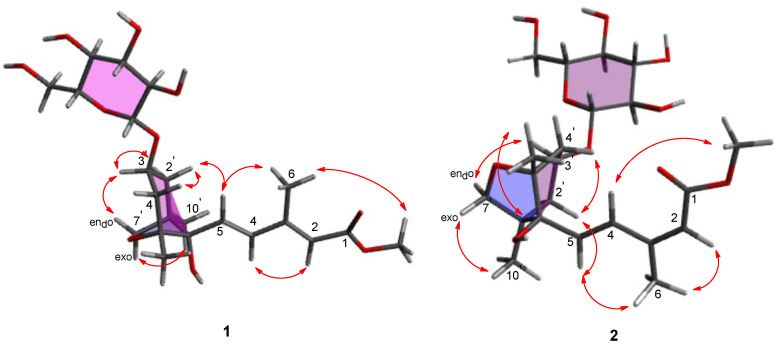
Key rotating frame Overhauser effect spectroscopy (ROESY) (arrows) correlations of compounds **1** and **2**.

The relative configuration of **1** was identified by rotating frame Overhauser effect spectroscopy (ROESY) (Figure S6) (Figure 3). The ROESY correlations between H-2’_ax_ and H-4’_ax_/H-5 proved that these protons are positioned on the same side (*β* axial) in the system of cyclohexane ring. Further, the correlation of the oxymethine proton H-3’ with H-2’_eq_/H-7’_endo_ and H-7’_exo_ with H-10’ was observed in the ROESY spectrum (Figure 3), which indicated that the oxymethine H-3’ proton are in the same side (*α* face) as an oxymethylene H-7’ in the cyclohexane ring. Thus, the relative configuration of **1** was unambiguously assigned as 1’R*,3’S*,5’R*,8’S*. In contrast, the geometry of the 3-methyl-penta-2,4-dienoic acid methyl ester group was determined by the analysis of the vicinal ^1^H coupling constant and the ROESY correlations (Figure 3). The olefin Δ^4/5^ in compound **1** was determined as a *trans*-conformation based on the vicinal ^1^H coupling constant (*J*_4,5_ = 15.5 Hz) [18]. The geometry of olefin Δ^2/3^ was assigned by comparing the results of ROESY analysis for compounds **1** and **2**. The ROESY spectrum of **1** did not have a correlation between H-2 and H-6, whereas the that of **2** displayed a correlation between H-2 and H-6. In addition, the ROESY correlations of H-5/H-6, H-2/H-4, and H-6/OCH_3_ were observed in compound **1,** whereas the ROESY correlations of H-5/H-6 and H-4/OCH_3_ were observed in compound **2** (Figure 3). These results provided strong evidence that compound **1** had 2*E* and 4*E* olefinic double bonds.

**Figure 4:**
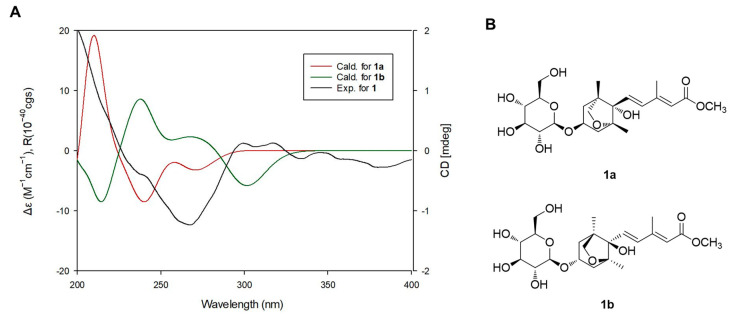
(**A**) Experimental and calculated electronic circular dichroism (ECD) spectra of compound **1** and (**B**) the structures of **1a** and **1b**.

LC/MS-UV-based method was performed to confirm the absolute configuration of the sugar unit [16] and enzymatic hydrolysis of **1** with hesperidinase resulted in the production of a glucopyranose. The absolute sugar configuration was confirmed as D- form by comparing the retention time of its thiocarbamoyl-thiazolidine derivative with the time of the standard sample (D-glucopyranose) from LC/MS analysis. The coupling constant (*J* = 8.0 Hz) of anomeric proton signal was typical of the *β*-form of glucopyranose [16], which indicated that the sugar unit is a *β*-D-glucopyranose. Finally, to verify the absolute configuration of **1**, two possible isomers, **1a** (2*E*,4*E*,1’*R*,3’*S*,5’*R*,8’*S*) and **1b** (2*E*,4*E*,1’*S*,3’*R*,5’*S*,8’*R*), were used for ECD calculations utilizing time-dependent-density-functional theory (TD-DFT) at the B3LYP/def2-TZVPP//B3LYP/def-SV(P) level for all atoms. The results showed that the ECD curve of **1a** (red line) was consistent with the experimental ECD spectrum of **1** (Figure 4). Thus, the absolute configuration of **1** was determined as 2*E*,4*E*,1’*R*,3’*S*,5’*R*,8’*S,* and the structure of **1** was elucidated as (2*E*,4*E*)-dihydrophaseic acid methyl ester-3-*O*-*β*-D-glucopyranoside. To the best of our knowledge, recently, free acid and glucoside ester of compound **1** were reported from *Annona glabra* fruit [19].

### 2.3. Inhibitory Effects of Compounds **1** and **2** on Adipogenesis in 3T3-L1 Preadipocytes

**Figure 5:**
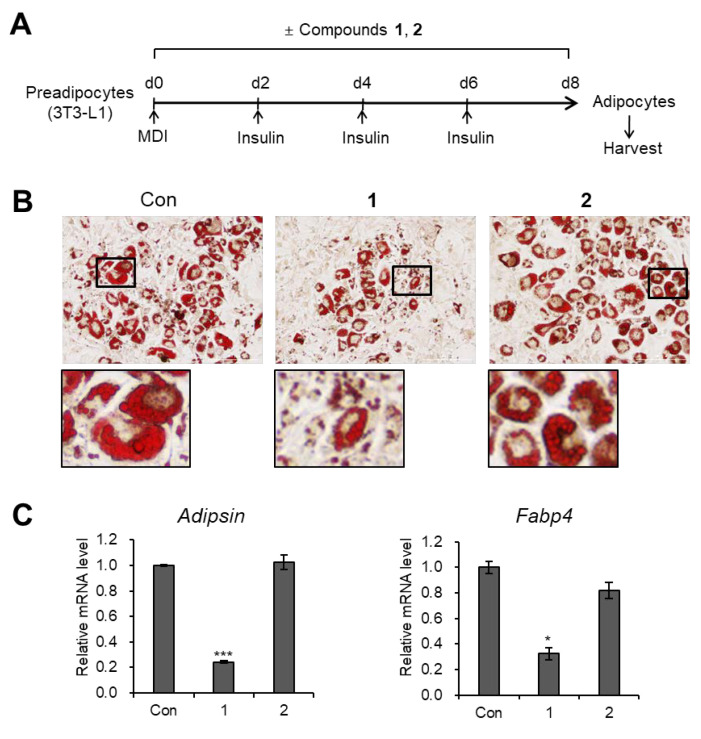
Inhibitory effects of compounds **1** and **2** on adipogenesis. (**A**) Schematic representation of the differentiation 3T3-L1 cells into adipocytes. Compounds **1** and **2** were used to treat the cells during the entire process of differentiation. (**B**) Oil Red O staining of 3T3-L1 adipocytes treated with compounds **1** and **2** (20 μM) during adipogenesis. (**C**) mRNA levels of the *Adipsin* and *Fabp4* in 3T3-L1 adipocytes treated with compounds **1** and **2** (20 μM) during adipogenesis. Data represent the means ± SEM for *n* = 3. * *p* < 0.05, *** *p* < 0.001.

The isolated compounds **1** and **2** were evaluated for their antiadipogenic properties [20,21,22], we independently treated 3T3-L1 cells with compounds **1** and **2** (20 μM, each) during adipogenesis (Figure 5A). Lipid droplets were visualized with Oil Red O staining, and the results showed that only compound **1** effectively inhibited *de novo* adipogenesis and lipid accumulation in adipocytes, whereas compound **2** did not alter the number of adipocytes or the size of lipid droplets (Figure 5B). Likely, the transcription levels of mature adipocyte marker genes (*Adipsin* and *Fabp4*) were significantly decreased upon exposure to compound **1**, but were not affected by compound **2** (Figure 5C). These data indicated that adipogenesis of preadipocytes is prevented only by the compound **1**.

## 3. Materials and Methods 

### 3.1. Plant Material

The florets of *C. tinctorius* were collected in Pocheon, Gyeonggi-do, Korea and purchased from Dongyang Pharm in September 2018. Detailed information is provided in the Supplementary Materials.

### 3.2. Extraction and Isolation

The florets of *C. tinctorius* (1.8 kg) were extracted using 80% aqueous MeOH (each 20 L × 2 days) at room temperature and filtered, and then combined and concentrated under vacuum pressure using a rotary evaporator, which gave a MeOH extract (530.0 g). The MeOH extract was divided in half, suspended in distilled water (700 mL, each), and then solvent partitioned with hexane, dichloromethane, ethyl acetate, and *n*-butanol. Four layers were solvent-partitioned, and hexane-soluble (37.6 g), CH_2_Cl_2_-soluble (3.3 g), EtOAc-soluble (10.4 g), and BuOH-soluble fractions (57.3 g) were obtained. After solvent-partition, the residue was solvent partitioned again with acetone, affording an acetone-soluble fraction (68.7 g). The EtOAc-soluble layer (10.4 g) was applied to silica gel column chromatography (300 g, eluted with CH_2_Cl_2_/MeOH (50:1→1:1, *v:v*) gradient solvent system, and washed with 90% MeOH) to yield seven fractions (E1‒E7). Fraction E5 (3.2 g) was separated by MPLC Yamazen UNIVERSAL Premium ODS-SM column with MeOH/H_2_O (30-100% MeOH) to yield five subfractions (E51‒E55). Subfraction E52 (638.7 mg) was subjected to Sephadex LH-20 with 100% MeOH to yield three subfractions (E521‒E523). Subfraction E521 (267.2 mg) was separated by preparative reversed-phase HPLC with a gradient solvent system of MeOH/H_2_O (50‒100% MeOH) to yield five subfractions (E5211‒E5215). Subfraction E5212 (87 mg) was subjected to silica gel column chromatography [3.0 g, eluted with CH_2_Cl_2_/MeOH (50:1→1:1) gradient solvent system, and washed with 90% MeOH] to yield five subfractions (E52121‒E52125). Subfraction E52124 (34.4 mg) was purified by semipreparative HPLC (35% MeOH) to yield compounds **2** (*t*_R_ 39.0 min, 2.6 mg) and **1** (*t*_R_ 39.5 min, 0.9 mg).

#### (2*E*,4*E*)-Dihydrophaseic Acid Methyl Ester-3-*O*-β-D-Glucopyranoside (**1**)

White, amorphous powder; ${\left[\alpha \right]}_{D}^{25}$− 10.0 (*c* = 0.03, MeOH); UV (MeOH) λ_max_ nm (log ε): 197 (1.17), 215 (0.64), 271 (3.45); IR (KBr) *ν*_max_ cm^-1^: 3302, 2915, 1698, 1615, 1452, 1040; ^1^H (850 MHz) and ^13^C (212.5 MHz) NMR data, see Table 1; ESIMS (positive-ion mode) *m/z* 481 [M+Na]^+^; HR-ESIMS (positive-ion mode) *m/z* 459.2220 [M + H]^+^ (calculated for C_22_H_35_O_10_ 459.2230).

### 3.3. Computational Analysis

To acquire the optimal conformation of **1a/1b,** computational DFT calculations were performed [23,24,25,26,27]. Detailed information is provided in the section of computational analysis in Supplementary Materials.

### 3.4. Enzymatic Hydrolysis and Absolute Configuration Determination of the Sugar Moiety

The absolute configuration of the sugar moiety was determined using an LC/MS-UV-based method [16]. Detailed information is provided in the section of enzymatic hydrolysis in Supplementary Materials.

### 3.5. Cell Culture and Differentiation

3T3-L1 preadipocytes, purchased from the American Type Culture Collection (ATCC® CL-173™), were grown in Dulbecco′s Modified Eagle′s Medium (DMEM) supplemented with 10% bovine calf serum and 1% penicillin/streptomycin (P/S). Detailed information is provided in the section of cell culture and differentiation in Supplementary Materials.

### 3.6. Oil Red O Staining

Oil Red O staining was conducted to visualize lipid droplets accumulated in adipocytes. Detailed information is provided in the section of Oil Red O staining in Supplementary Materials.

### 3.7. Reverse Transcription and Quantitative Real-Time PCR

To detect RNA expression, total RNA was extracted from adipocytes utilizing Easy-Blue reagent (Intron Biotechnology). The detailed information is provided in the section of reverse transcription and quantitative real-time PCR in Supplementary Materials. The sequences of the qPCR primers were as followed:

*β-actin* forward, 5′-ACGGCCAGGTCATCACTATTG-3′
*β-actin* reverse, 5′-TGGATGCCACAGGATTCCA-3′
*Adipsin* forward, 5′-CATGCTCGGCCCTACATG-3′
*Adipsin* reverse, 5′-CACAGAGTCGTCATCCGTCAC-3′
*Fabp4* forward, 5′-AAGGTGAAGAGCATCATAACCCT-3′
*Fabp4* reverse, 5′-TCACGCCTTTCATAACACATTCC-3′

### 3.8. Statistical Analysis

The significance of the results was evaluated using two-tailed Student’s *t*-test in MS Excel. Significance was judged based on the *p*-value. Data represent the mean ± SEM for *n* = 3. * *p* < 0.05, ** *p* < 0.01, *** *p* < 0.001.

## 4. Conclusions

To conclude, this study elaborated on the phytochemical analysis of the MeOH extracts of the florets of *C. tinctorius* and the isolation of two dihydrophaseic acid glucosides (**1** and **2**). Compound **1** was configured as (2*E*,4*E*)-dihydrophaseic acid methyl ester-3-*O*-*β*-D-glucopyranoside, and the absolute configurations of **1** were elucidated by quantum chemical ECD calculations and enzymatic hydrolysis. Compound **1** was identified to be a geometric isomer of **2**. The antiadipogenic effects of the isolated compounds (**1** and **2**) were evaluated by lipid droplet staining and by determining the expression of adipogenic genes. Among the two, only compound **1** could effectively inhibit adipocyte generation. This result therefore provides a potential therapeutic strategy to prevent adipogenesis in obesity. 

## Figures and Tables

**Table 1:** ^1^H and ^13^C NMR data of compounds **1** and **2** in CD_3_OD (δ in ppm, 850 MHz for ^1^H and 212.5 MHz for ^13^C) ^a^.

Position	1		2	
	*δ*_H_	*δ*_C_	*δ*_H_	*δ*_C_
1		168.8		167.9
2	5.89 s	120.0	5.78 s	117.3
3		153.2		151.9
4	6.66 d (15.5)	137.3	8.01 d (15.5)	131.5
5	6.58 d (15.5)	134.2	6.55 d (15.5)	135.4
6	2.34 s	13.9	2.09 s	20.8
1’		49.4		49.2
2’	*ax*: 1.80 dd (14.0, 10.0); *eq*: 2.00 ddd (14.0, 7.0, 1.5)	42.5	*ax*: 1.81 dd (13.5, 11.0); *eq*: 2.19 dd (13.5, 5.5)	42.3
3’	4.26 tt (10.0, 7.0)	73.6	4.28 tt (11.0, 5.5)	73.6
4’	*ax*: 1.80 dd (14.0, 10.0); *eq*: 2.20 ddd (14.0, 7.0, 1.5)	42.5	*ax*: 1.81 m; *eq*: 1.99 dd (13.5, 5.5)	42.3
5’		87.6		87.2
7’	*endo*: 3.77 d (7.5); *exo*: 3.80 d (7.5)	76.9	*endo*: 3.76 d (7.5); *exo*: 3.81 d (7.5)	76.7
8’		83.1		83.1
9’	1.13 s	19.4	1.17 s	19.2
10’	0.91 s	16.0	0.94 s	15.9
1’’	4.37 d (8.0)	102.1	4.36 d (8.0)	102.5
2’’	3.14 dd (9.0, 8.0)	74.9	3.14 dd (9.0, 8.0)	74.9
3’’	3.28 m	77.8	3.28 m	77.8
4’’	3.28 m	71.3	3.28 m	71.3
5’’	3.35 m	77.9	3.35 m	77.9
6’’	3.86 dd (12.0, 2.0); 3.66 dd (12.0, 5.5)	62.6	3.86 dd (12.0, 2.0); 3.66 dd (12.0, 5.5)	62.3
OCH_3_’	3.70 s	51.2	3.70 s	50.8

^a^*J*-values are expressed in parentheses and reported in Hz; ^13^C NMR assignments are based on HSQC, HMBC, and ^1^H-^1^H COSY experiments; ax: axial and eq: equatorial.

## Supplementary Materials

The following are available online at https://www.mdpi.com/2223-7747/9/7/858/s1, General experimental procedures, 1D and 2D NMR, and HR-ESIMS of compound **1** are available online free of charge. Figure S1: The HRESIMS data of **1**, Figure S2: The ^1^H NMR spectrum of **1** (CD_3_OD, 850 MHz), Figure S3: The ^1^H-^1^H COSY spectrum of **1**, Figure S4: The HSQC spectrum of **1**, Figure S5: The HMBC spectrum of **1**, Figure S6: The ROESY spectrum of **1**.
