# Supplementary material for: Discovery of Dihydrophaseic Acid Glucosides from the Florets of Carthamus tinctorius
Source: Plants (Basel). 2020 Jul 7;9(7):858. doi: 10.3390/plants9070858 (PMC7412175; doi:10.3390/plants9070858)

Supplementary Materials

Discovery of Dihydrophaseic Acid Glucosides from the Florets of *Carthamus tinctorius*

Su Cheol Baek[1,†], Bum Soo Lee[1,†], Sang Ah Yi[1], Jae Sik Yu[1], Jaecheol Lee[1], Yoon-Joo Ko[2], Changhyun Pang[3,*] and Ki Hyun Kim[1,*]

[1]School of Pharmacy, Sungkyunkwan University, Suwon 16419, Republic of Korea; schii513@daum.net (S.C.B.); kosboybs@naver.com (B.S.L.); angelna1023@hanmail.net (S.A.Y.); jsyu@bu.edu (J.S.Y.); jaecheol@skku.edu (J.L.).

[2]Laboratory of Nuclear Magnetic Resonance, National Center for Inter-University Research Facilities (NCIRF), Seoul National University, Gwanak-gu, Seoul 08826, Republic of Korea; yjko@snu.ac.kr (Y.-J.K.)

[3]School of Chemical Engineering, Sungkyunkwan University, Suwon 16419, Republic of Korea

[*]Correspondence: chpang@skku.edu (C.P.); khkim83@skku.edu (K.H.K.); Tel.: +82-031-290-7341 (C.P.); +82-31-290-7700 (K.H.K.)

[†]These authors contributed equally to this work

Supporting Information Contents:

General experimental procedures …… S3
Figure S1. The HRESIMS data of **1** …… S7
Figure S2. The ^{1}H NMR spectrum of **1** (CD_3OD, 850 MHz) …… S8
Figure S3. The ^{1}H-^{1}H COSY spectrum of **1** …… S9
Figure S4. The HSQC spectrum of **1** …… S10
Figure S5. The HMBC spectrum of **1** …… S11
Figure S6. The ROESY spectrum of **1** …… S12

General experimental procedures

Optical rotations were measured using a Jasco P-2000 polarimeter (Jasco, Easton, MD, USA). Infrared (IR) spectra were recorded with a Bruker IFS-66/S FT-IR spectrometer (Bruker, Karlsruhe, Germany). Ultraviolet (UV) spectra were acquired on an Agilent 8453 UV-visible spectrophotometer (Agilent Technologies, Santa Clara, CA, USA). Nuclear magnetic resonance (NMR) spectra were recorded with a Bruker AVANCE III HD 850 NMR spectrometer with a 5 mm TCI CryoProbe operating at 850 MHz (^{1}H) and 212.5 MHz (^{13}C) (Bruker, Karlsruhe, Germany), with chemical shifts given in ppm (δ) for ^{1}H and ^{13}C NMR analyses. Medium-pressure liquid chromatography (MPLC) was performed with a Smart Flash AKROS (Yamazen, Osaka, Japan). Preparative high-performance liquid chromatography (HPLC) was performed using a Waters 1525 Binary HPLC pump with a Waters 996 Photodiode Array Detector (Waters Corporation, Milford, CT, USA) and an Agilent Eclipse C18 column (250 × 21.2 mm, 5 μm; flow rate: 5 mL/min; Agilent Technologies, Santa Clara, CA, USA). Semi-preparative HPLC was performed using a Shimadzu Prominence HPLC System with SPD-20A/20AV Series Prominence HPLC UV-Vis detectors (Shimadzu, Tokyo, Japan) and a Phenomenex Luna C18 column (250 × 10 mm, 5 μm; flow rate: 2 mL/min; Phenomenex, Torrance, CA, USA). LC/MS analysis was performed on an Agilent 1200 Series HPLC system equipped with a diode array detector and 6130 Series ESI mass spectrometer using an analytical Kinetex C18 100 Å column (100 × 2.1 mm, 5 μm; flow rate: 0.3 mL/min; Phenomenex, Torrance, CA, USA). All HR-ESI-MS data were obtained with an Agilent 6545 Q-TOF LC/MS spectrometer (Agilent Technologies, Santa Clara, CA, USA). Silica gel 60 (230-400 mesh; Merck, Darmstadt, Germany) and RP-C_{18} silica gel (Merck, 230-400 mesh) were used for column chromatography. The packing material for molecular sieve column chromatography was Sephadex LH-20 (Pharmacia, Uppsala, Sweden). Merck precoated silica gel F_{254} plates and RP-C_{18} F_{254s} plates were used for thin-layer chromatography (TLC). Spots were detected after TLC under UV light or by heating after spraying with anisaldehyde-sulfuric acid. The 3D molecular modeling was performed by using ChemBioDraw Ultra and Avogadro.

Plant material

The florets of *C. tinctorius* were collected in Pocheon, Gyeonggi-do, Korea and purchased from Dongyangpharm in September 2018. The

plant was identified by one of the authors (K. H. Kim). A voucher specimen (HH-18-12) was deposited in the herbarium of the School of Pharmacy, Sungkyunkwan University, Suwon, Korea.

Computational analysis

To acquire the optimal conformation of **1a/1b,** computational DFT calculations were performed. The first structural energy minimization of **1a/1b** was performed by utilizing Avogadro 1.2.0 with a UFF force field. The ground state geometries of **1a/1b** were then established by Tmolex 4.3.1 with the DFT settings of B3-LYP functional/M3 grid size, geometry optimization options of energy 10^{-6} hartree, gradient norm $|dE/dxyz| = 10^{-3}$ hartree/bohr, and the basis set def-SV(P) for all atoms [23-26]. The calculated ECD spectra of the optimized structures were acquired at the B3LYP/DFT functional settings with the basis set def2-TZVPP for all atoms [23-27]. The obtained ECD spectra were simulated by overlying each transition, where σ is the width of the band at height 1/e; and ΔE_i and R_i are the excitation energies and rotatory strengths for transition *i*, respectively. In the present study, the value of σ was 0.10 eV.

$$\Delta\epsilon(E) = \frac{1}{2.297 \times 10^{-39}} \frac{1}{\sqrt{2\pi\sigma}} \sum_{A}^{i} \Delta E_i R_i e^{[-(E-\Delta E_i)^2/(2\sigma)^2]}$$

Enzymatic hydrolysis and absolute configuration determination of the sugar moiety

The absolute configuration of the sugar moiety was determined using an LC/MS-UV-based method [16]. Compound **1** (0.3 mg) was hydrolyzed with crude hesperidinase (10 mg, from *Aspergillus niger*; Sigma-Aldrich) at 37°C for 72 h, and EtOAc was used for the extraction. The aqueous layer was evaporated using a vacuum evaporator and dissolved in anhydrous pyridine (0.5 mL) with the addition of L-cysteine methyl ester hydrochloride (1.0 mg). After the reaction mixture was heated at 60°C for 1 h, *o*-tolylisothiocyanate (50 μL) was added, and the mixture was incubated at 60°C for 1 h. The reaction product was evaporated using a vacuum evaporator and dissolved in MeOH. After that, the dissolved reaction product was directly analyzed by LC/MS [MeOH/H_2O, 1:9 → 7:3 gradient system (0-30 min), 100% MeOH (31-41 min), 0% MeOH (42-52 min); 0.3 mL/min] using analytical Kinetex C18 100 Å column (100 mm × 2.1 mm i.d., 5 μm). The sugar moiety

from **1** was identified as D-glucopyranose based on the comparison of the retention time with an authentic sample (t_R: D-glucopyranose 19.3 min).

Cell culture and differentiation

3T3-L1 preadipocytes, purchased from the American Type Culture Collection (ATCC® CL-173™), were grown in Dulbecco′s Modified Eagle′s Medium (DMEM) supplemented with 10% bovine calf serum and 1% penicillin/streptomycin (P/S). For the differentiation of 3T3-L1 cells into mature adipocytes, the cells were cultured in DMEM supplemented with 10% FBS, 1% P/S, 0.5 mM 3-isobutyl-1-methylxanthine, 1 μM dexamethasone, and 1 μg/mL insulin (day 0). Next, the medium was replaced every other day with DMEM containing 10% FBS, 1% P/S, and 1 μg/mL insulin. To assess the effects of compounds **1** and **2** on adipogenesis, we treated 3T3-L1 cells with compounds **1** and **2** during the entire process of adipogenesis. At day 8, the cells were harvested and subjected to further experiments, including immunoblotting or reverse transcription (RT)-quantitative PCR (qPCR).

Oil Red O staining

Oil Red O staining was conducted to visualize lipid droplets accumulated in adipocytes. Mature adipocytes were fixed with 10% formaldehyde for 1 h and washed with 60% isopropanol. Next, the cells were incubated with Oil Red O working solution for 1 h, and then washed twice with distilled water. To prepare Oil Red O stock solution, 300 mg of Oil Red O powder was dissolved in 100 mL of 99% isopropanol. The Oil Red O working solution, containing three parts of Oil Red O stock solution and two parts of distilled water, was prepared just before use.

Reverse transcription and quantitative real-time PCR

To detect RNA expression, total RNA was extracted from adipocytes utilizing Easy-Blue reagent (Intron Biotechnology). cDNA was generated by subjecting 1 μg of total RNA to reverse transcription using a Maxim RT-PreMix Kit (Intron Biotechnology). Next, qPCR was performed by mixing cDNA, KAPA SYBR® FAST qPCR Master Mix (Kapa Biosystems), and gene-specific primers. The signal generated during qPCR was detected using CFX96 TouchTM or Chromo4TM real-time PCR detector (Bio-Rad). Relative mRNA levels were

normalized to those of β-actin for each reaction. The sequences of the qPCR primers were as follows:

β-actin forward, 5′-ACGGCCAGGTCATCACTATTG-3′

β-actin reverse, 5′-TGGATGCCACAGGATTCCA-3′

Adipsin forward, 5′-CATGCTCGGCCCTACATG-3′

Adipsin reverse, 5′-CACAGAGTCGTCATCCGTCAC-3′

Fabp4 forward, 5′-AAGGTGAAGAGCATCATAACCCT-3′

Fabp4 reverse, 5′-TCACGCCTTTCATAACACATTCC-3′

Figure S1. The HRESIMS data of **1**

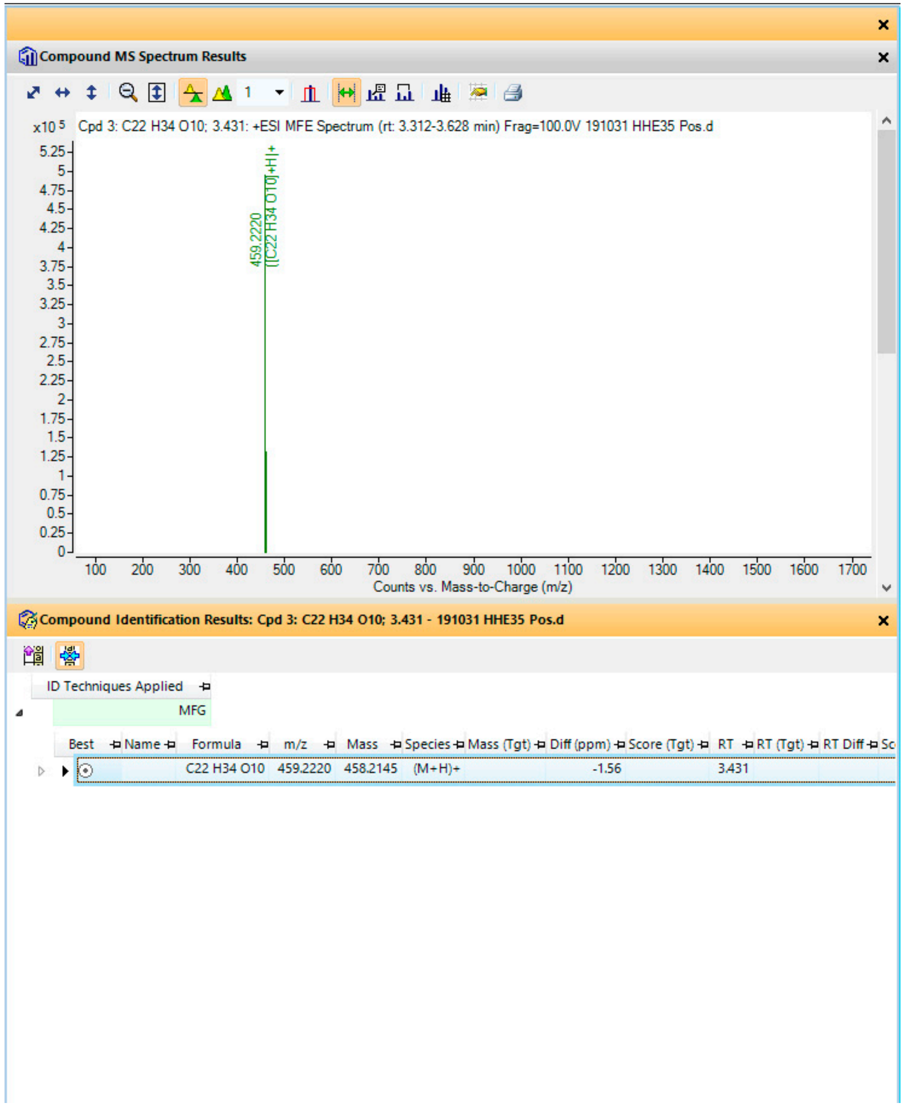

Figure S2. The ^{1}H NMR spectrum of **1** (CD_3OD, 850 MHz)

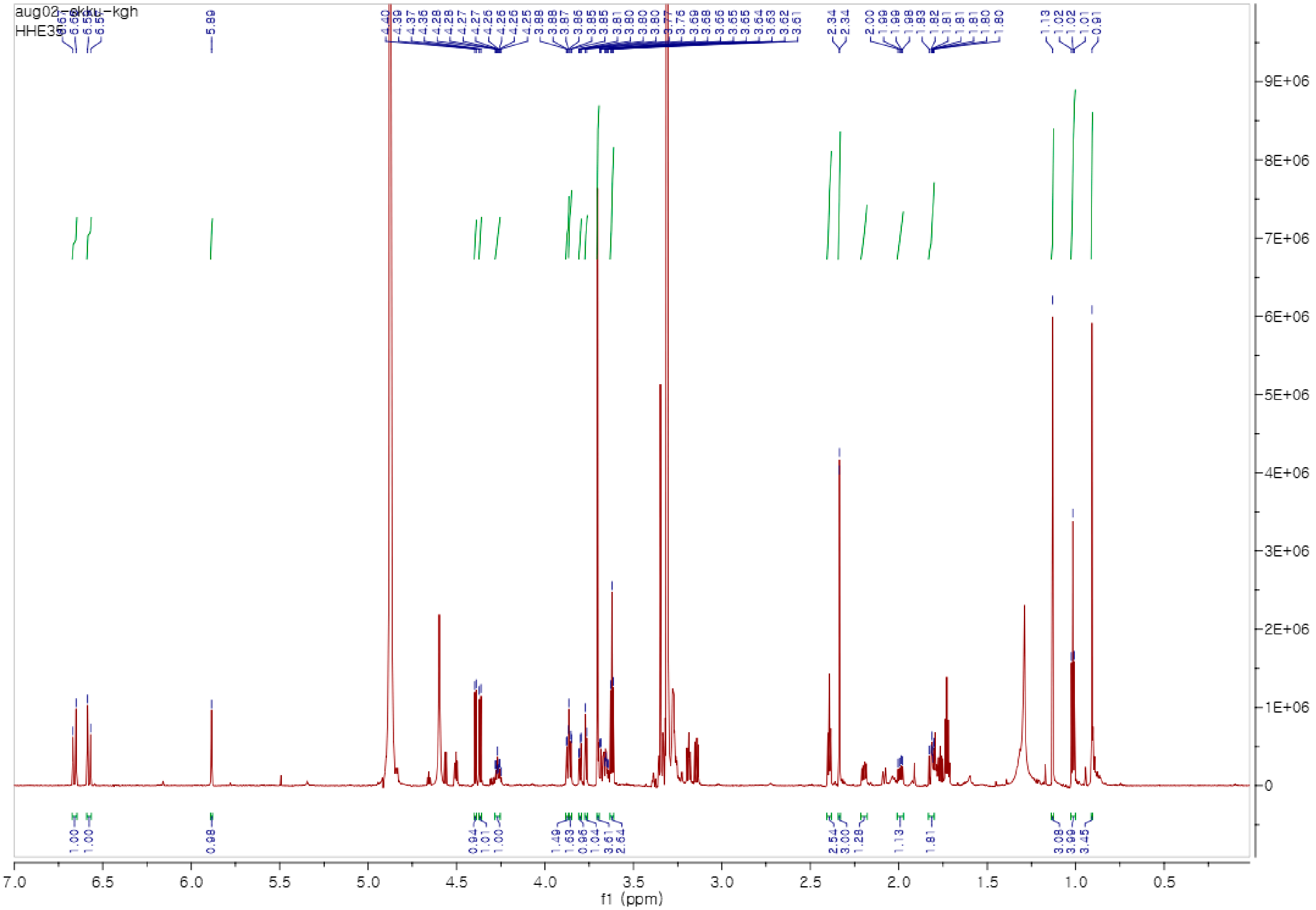

Figure S3. The ^{1}H-^{1}H COSY spectrum of **1**

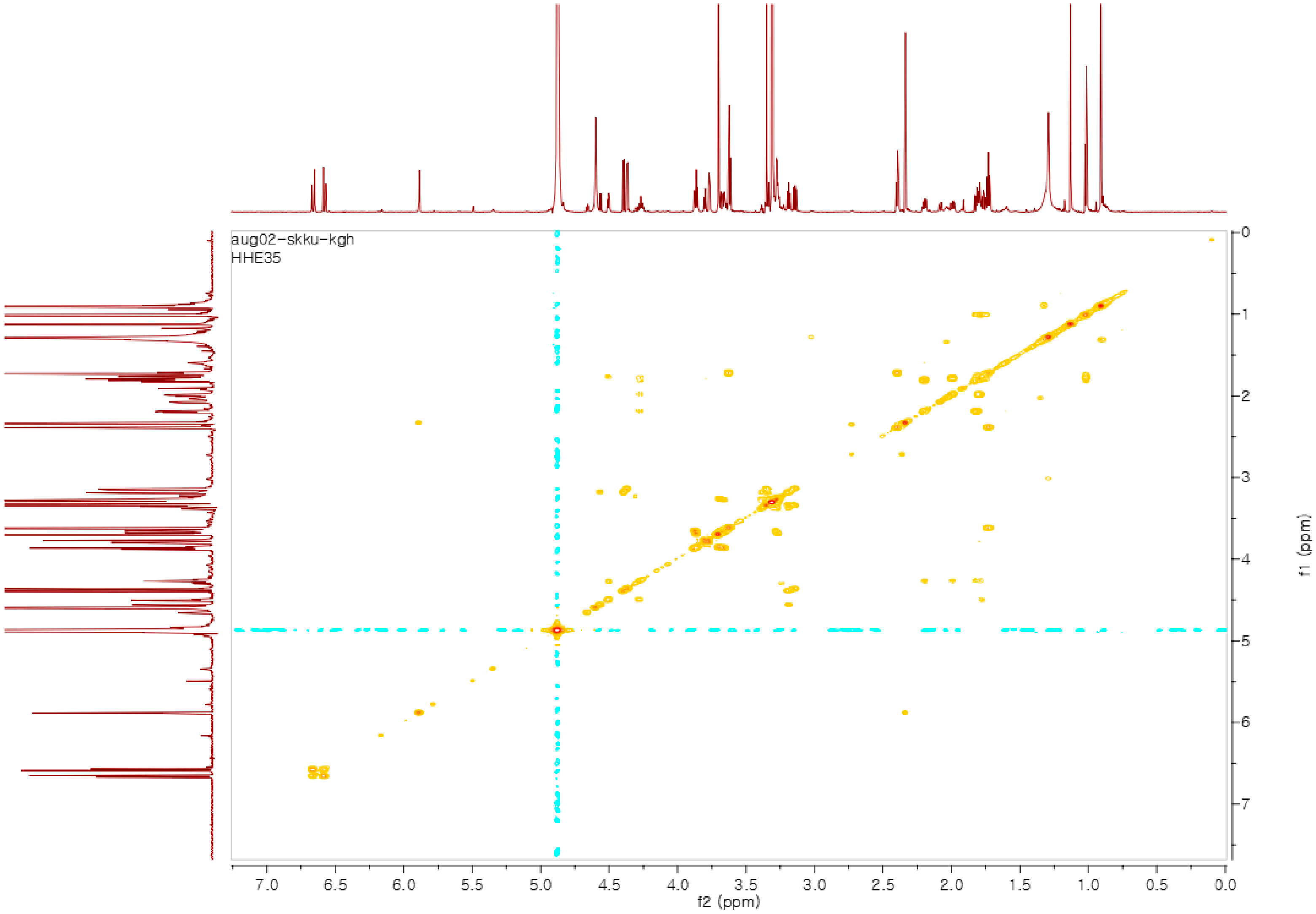

Figure S4. The HSQC spectrum of **1**

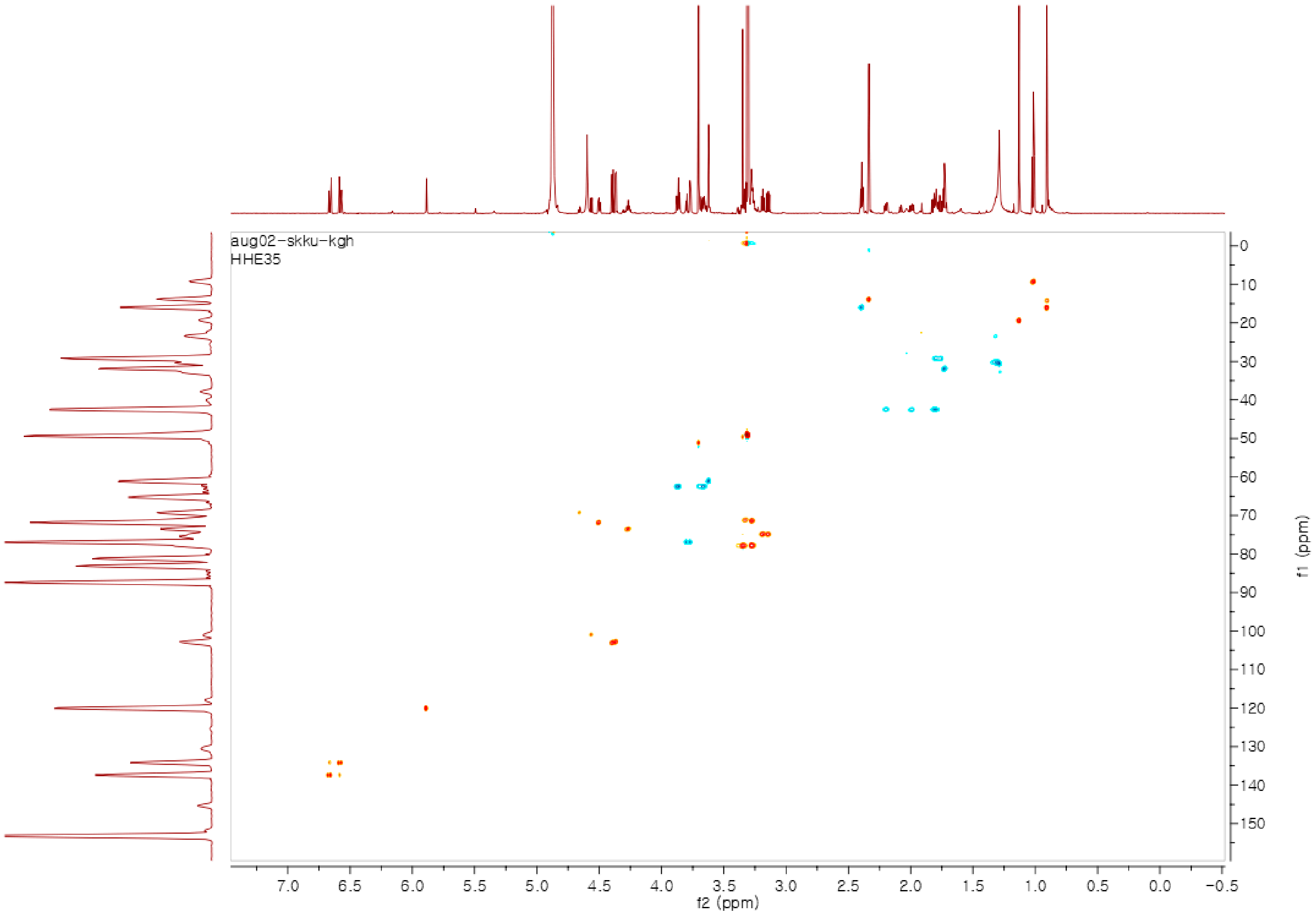

Figure S5. The HMBC spectrum of **1**

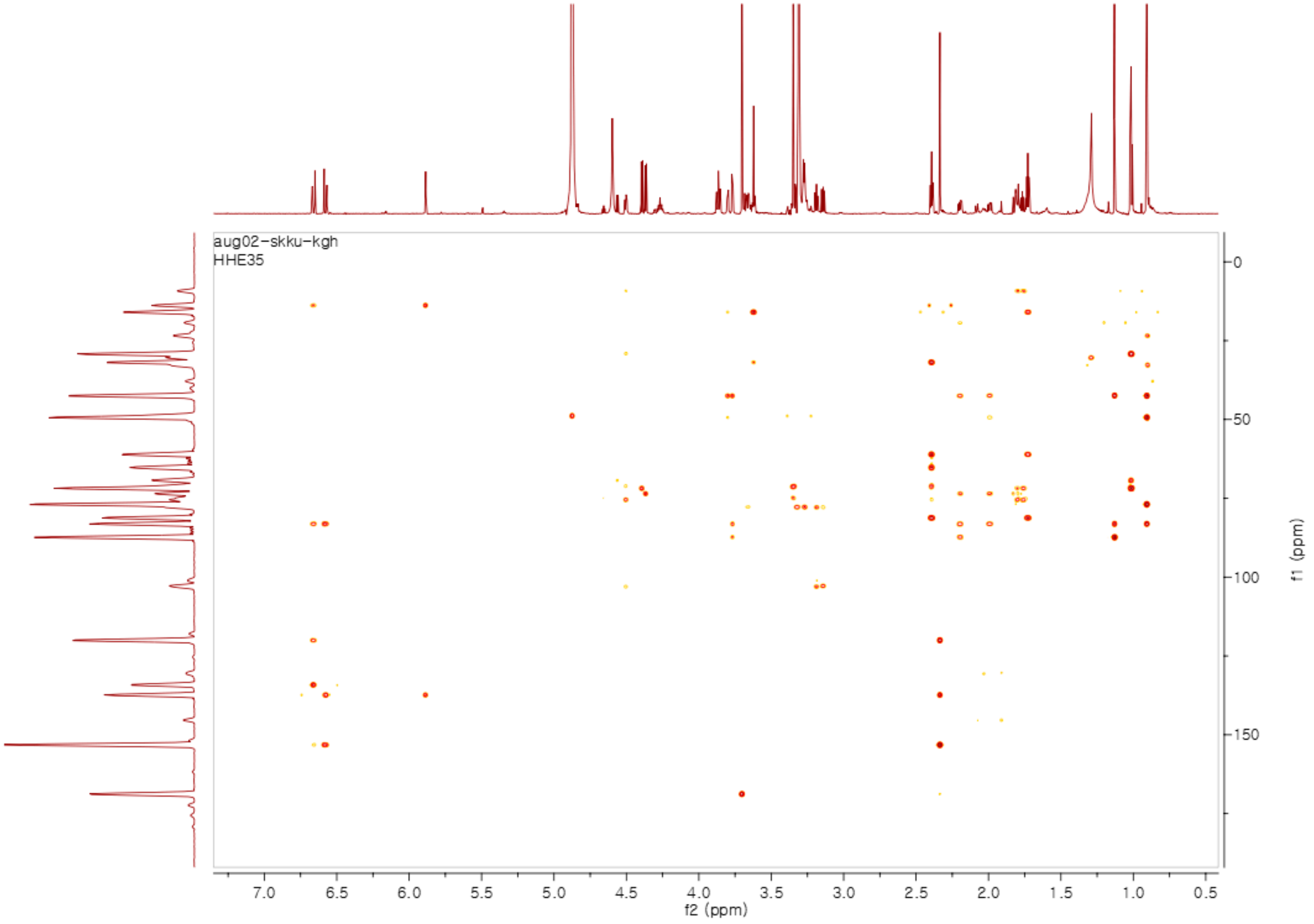

Figure S6. The ROESY spectrum of **1**

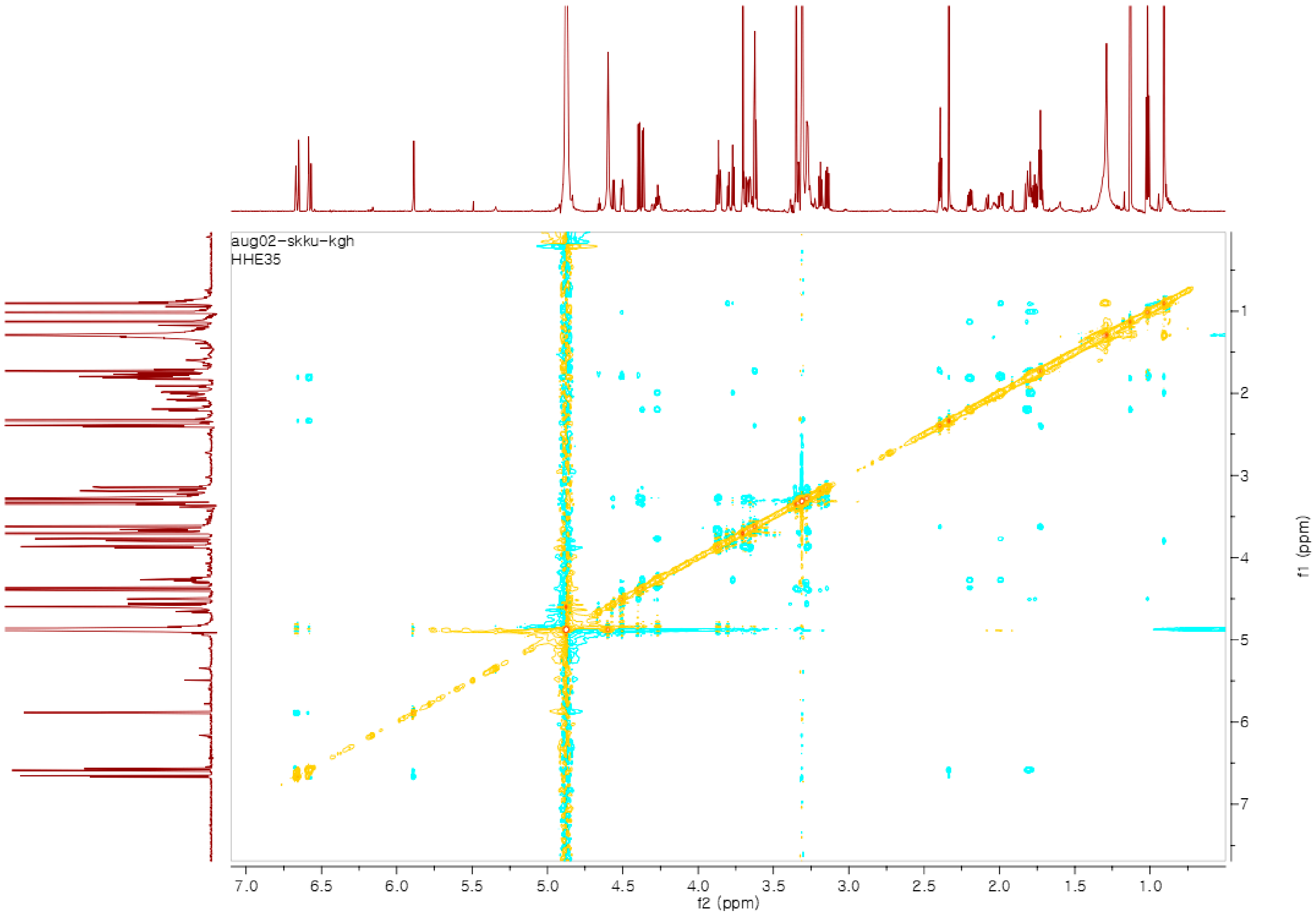

Supplement: Supplementary file 1 [file plants-09-00858-s001.pdf]
